# A Simple and Effective Mechanical Method for Adipose-Derived Stromal Vascular Fraction Isolation

**DOI:** 10.7759/cureus.57137

**Published:** 2024-03-28

**Authors:** Patroklos Goulas, Maria Karakwta, Apostolos Zatagias, Maria Bakoutsi, Alexandros Zevgaridis, Aristeidis Ioannidis, Despoina Krokou, Antonios Michalopoulos, Vasileios Zevgaridis, George Koliakos

**Affiliations:** 1 1st Propedeutic Surgical Department, American Hellenic Educational Progressive Association (AHEPA) University Hospital, Aristotle University of Thessaloniki, Thessaloniki, GRC; 2 Laboratory of Biological Chemistry, School of Medicine, Aristotle University of Thessaloniki, Thessaloniki, GRC; 3 Department of Surgery, Interbalkan Medical Center, Thessaloniki, GRC; 4 Department of Surgery, American Hellenic Educational Progressive Association (AHEPA) University Hospital, Thessaloniki, GRC; 5 1st Surgical Department, Aristotle University of Thessaloniki, Thessaloniki, GRC

**Keywords:** mechanical isolation, lipoaspirate, stomal vascular fraction, adipose-derived stem cells, mesenchymal stem cells

## Abstract

Over the last decades, there has been ongoing and evolving research concerning regenerative medicine, specifically, stem cells. The most common source of adult mesenchymal stem cells (MSCs) remains the adipose tissue and the easiest way to obtain such tissue is lipoaspirate. The fatty tissue obtained can be processed either in an enzymatic way, which is time-consuming and expensive and carries several dangers for the viability of the stem cells included, or with mechanical means which are fast, inexpensive, yield enough viable cells, and can be readily used for autologous transplantation in one-stage procedures.

Herein, we demonstrate our non-enzymatic method for obtaining adipose-derived stromal vascular fraction comprising MSCs. The stromal vascular fraction was isolated via centrifugation, and the characteristics and numbers of the cells isolated have been tested with flow cytometry assay, cell culture, and differentiation.

Over 91% of viable MSCs were isolated using the mechanical method. The cells retained the ability to differentiate into osteocytes, adipocytes, and chondrocytes.

The method presented is simple, requiring no special equipment, and yields a viable population of stem cells in large numbers. These cells can be readily used in several operations (orthopedic, dentistry, fistulas, etc.) making feasible “one-stage” procedures, thus proving their benefits for the patient and the health care system.

## Introduction

Over the last two decades, there has been evolving and rapidly growing research on mesenchymal stem cells (MSCs), irrespective of the source of origin, meaning adipose, bone marrow, or placenta-derived stem cells. Publications regarding the tissue of origin, the isolation, the manipulation, and finally the clinical use of stem cells are becoming more and more frequent and are gaining the attention of both the laboratory researchers and the clinicians (surgeons, dentists, orthopedics, etc.). This whole new field of investigation and clinical practice has composed the concept of regenerative medicine, creating promises that, sometimes, could hardly be fulfilled. Stem cells have been under the spotlight due to some of their magnificent capabilities. MSCs are pluripotent, meaning that they can undergo self-renewal and multilineage differentiation into relevant cell populations. Although these cells were initially believed to be able to differentiate only into the tissue of origin, nowadays it is proven that they can differentiate into cells of all three germ layers (mesoderm, endoderm, and ectoderm) [1-5]. This characteristic makes MSCs so important for regenerative medicine as they can be used to heal a wide range of tissues. As mentioned before, the source of stem cells could be in several tissues. The tissue that includes plenty of stem cells and lies in abundance in the human body, is adipose tissue. Moreover, adipose tissue is easy to obtain and process, with the subcutaneous fat being ideal for this purpose via the well-established method of liposuction. Stromal vascular fraction (SVF) stands for a component of the lipoaspirate, which is a heterogeneous population of cells. In particular, it is the component of the lipoaspirate that precipitates to the bottom after the centrifugation of the lipoaspirate, having the adipose cells, connective tissue, and blood removed. This heterogeneous population includes cells with progenitor activity such as preadipocytes, MSCs, pericytes, endothelial cells, and macrophages. Most of these cells have an immunomodulatory role through cytokines and growth factors, thus providing a healing capacity to this fraction of the lipoaspirate [6-9]. Moreover, MSCs have further immunomodulatory abilities, such as downregulating immune responses and promoting tissue healing [10]. Until today, many studies have been published on the therapeutic role of MSCs in the repair of various injuries. Bearing all these in mind, contemporary regenerative medicine applications are in chase of finding the best means of delivering the largest amount of viable MSCs into a “cell-friendly” matrix, natural or artificial, with SVF being probably the most suitable one. This “holy grail” of regenerative medicine could be accomplished by various ways of obtaining and processing the MSCs. There is abundant literature, sometimes with conflicting results, presenting many different techniques that try to find the best source and method of isolating and processing the MSCs. Liposuction remains the gold standard for the collection of human adipose tissue [11,12] and as a result the best method for the isolation of human adipose-derived stromal stem cells (hASCs).

In this study, we isolated the adipose-derived SVF from lipoaspirates with a low-cost, non-enzymatic mechanical method. As a result, a large amount of viable and autogenous ASCs is delivered with a fast and cost-effective method. The advantage of our method is its simplicity, reproducibility, and cost-effectiveness in order to prepare a large amount of stem cells that are ready to use in one-stage surgical procedures such as perianal fistulas or chronic wound surgery.

## Technical report

Adipose tissue was obtained from abdominal subcutaneous lipoaspirates. The stromal vascular fraction was isolated via centrifugation following mechanical processing and was subsequently cultured, characterized, and differentiated in order to prove mesenchyme cell viability and pluripotency.

Materials

The materials used for this study are as follows: Dulbecco’s Modified Eagle’s Medium (DMEM)- High glucose (Biowest), StemPro Adipocyte Differentiation Basal medium (Gibco), StemPro Osteocyte/Chondrocyte Differentiation Basal medium (Gibco), Anti-Hu CD90 FITC (EXBIO), Anti-Hu CD105 PE (EXBIO), 7-AAD Viability Dye (IMMUNOTECH S.A.S, Beckman Coulter), Alizarin Red S (Sigma Aldrich), Alcian blue (Sigma Aldrich), and Oil Red O (Sigma Aldrich).

Adipose-derived SVF isolation 

Adipose tissue was obtained from patients who were scheduled to undergo abdominal surgical procedures for therapeutic reasons (i.e. laparotomy) after written consent from the patient according to the protocol approved by the bioethics committee of the Aristotle University of Thessaloniki (protocol number 370-9/ 10.5.2017). The lipoaspirate took place before the main operation, without having any impact on the patient’s outcome. The procedure was performed under general anesthesia with the patient in the supine position. Initially, the subcutaneous tissue of the abdominal wall was infiltrated with 300-400 ml of a solution consisting of 1 liter Ringer’s lactate, 40 ml lidocaine, and 1 mg adrenaline, using a 1 mm infiltration cannula. Consequently, a 3 or 4 mm blunt-tipped liposuction cannula, attached to a closed suction system of continuous negative pressure with a collection canister, was used to collect the fatty tissue of the abdominal wall. At the end of the procedure, which lasted about 10 minutes, a total amount of 300-400 ml of lipoaspirate was obtained, including adipose tissue, connective tissue, blood, and the infiltrating solution. The mixture collected was then placed in a simple sieve and was washed thoroughly with 1-2 liters of normal saline to remove red blood cells and the infiltrating solution, yielding about 100 ml of clean adipose tissue with few parts of connective tissue. The fat was further minced with Metzenbaum scissors and then transferred to a 10 ml Luer-lock syringe connected via a three-way stopcock with another empty 10 ml syringe. Further mincing with scissors was essential so the stringy parts of connective tissue that had been collected through the liposuction cannula could be chopped into smaller parts that would not block the flow of the processed specimen as described later on. The fat was shifted mildly and repeatedly between these two syringes about 30 times, thus emulsifying the tissue. The processed fat was transferred to a 15 ml Falcon tube adding 3 ml of normal saline. The saline was added in order to make a clear liquid phase after the centrifugation between the cell's sediment, mainly consisting of stem cells and the adipose cells above. This separating layer made the collection of the sediment easier and prevented the mixing of the cells to be collected with parts of connective tissue that weren’t completely separated. The tube was centrifuged in 558 g for 10 minutes and the SVF was collected with a spinal needle attached to a 5 ml syringe from the lower part of the tube (around 3 ml), with the cell pellet (containing the adipose-derived stem cells) forming a whitish precipitate, visible at the bottom of the tube. The pellet was resuspended in the appropriate medium for the characterization of cells with flow cytometry and cultivation.

Isolation of stem cells was also done with the enzymatic method to compare the effectiveness of the above method. Adipose tissue was washed with phosphate-buffered saline (PBS), minced, and digested with 1 mg/ml collagenase type-1 (Sigma) for 1 h at 37°C with constant shaking. After one hour, the sample was centrifuged for 10 min at 600 × g. The cellular pellet was resuspended in PBS and stained with CD-90, CD-105 antibodies, and 7-ADD Viability Dye for characterization with flow cytometry. 

Flow cytometry assay

The isolated cells were incubated with 20 μl CD90-FITC and 20 μl CD105-PE antibodies against MSC markers. 20 μl 7-AAD Viability Dye was added to determine the viability of the isolated cells. The cells were incubated with antibodies for 20 minutes at room temperature in the dark and after staining they were measured in a FACS Calibure (TM) flow cytometer (Becton Dickinson).

Cell culture and differentiation

After characterization, the remaining cells were cultivated in DMEM medium, supplemented with 10% FBS, and 1% Penicillin/ Streptomycin, in six-well plates at 37^o^C, under 5% CO_2_, and the culture medium was changed every 2-3 days. The cells were let to proliferate until they coated the surfaces of six-well plates by 60%. At this point, the cells were cultivated in adipogenic, osteogenic, and chondrogenic differentiation mediums for 30 days at the same conditions as described previously. The cultivation mediums were changing every two days. At 30 days, the terminal differentiation was monitored by Oil Red O, Alizarine Red S, and Alcian blue staining, respectively. 

**Figure 1:**
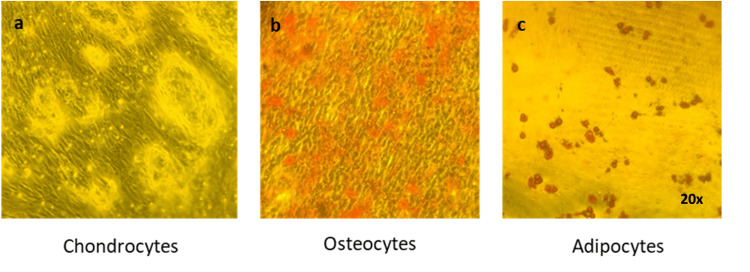
Chondrogenic, osteogenic, and adipogenic differentiation of ADSCs isolated by the mechanical method. (a) Alcian blue staining, (b) Alizarin red staining, and (c) Oil Red O staining were used to assess chondrogenic, osteogenic, and adipogenic differentiation, respectively. Representative images were taken at 20x magnification.

After the non-enzymatic isolation method, a mean of 59 x 10^6 adipose tissue-derived stem cells (ADSCs) was collected from 20 ml of lipoaspirate. Ninety-one percent of the isolated cells were found to be viable after staining with 7-AAD. Also, the isolated cells were characterized by flow cytometry assay, using the CD90 and CD105 antibodies as mesenchymal markers. Eighty-nine percent of the isolated cells co-expressed the CD90 and CD105 surface markers (Figure 1). On the other hand, the cell population isolated using collagenase had the same viability as the cells isolated with the mechanical method but a higher percentage of double positives CD-90/CD-105 cells (almost 91%). 

**Figure 2:**
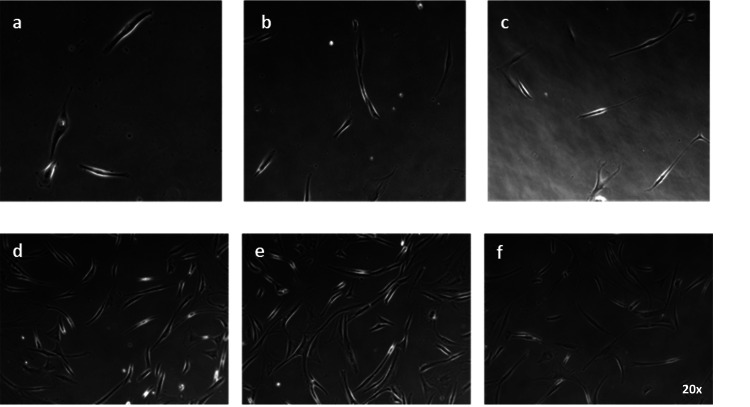
Adipose-derived mesenchymal stem cells in culture isolated by the mechanical method. (a-c) Ιsolated cells after two days in culture and (d-f) isolated cells after 25 days in culture. The cells have spindle-like morphology resembling adipose mesenchymal stem cells. Representative images were taken at 20x magnification. The cells have spindle-like morphology resembling adipose mesenchymal stem cells. As it was expected, the second sample was much larger due to the longer incubation period.

**Figure 3:**
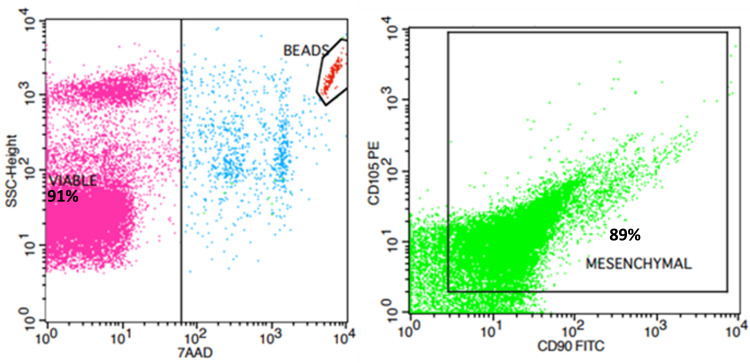
Characterization of the cells isolated by the mechanical method by flow cytometry. The cells are positive for CD90 and CD105 markers with over 90% viability. 89% of the isolated cells co-expressed the CD90 and CD105 surface markers.

The ADSCs were cultured at 37^o^C, under 5% CO_2_ (Figure 2), having a typical fibroblast-like morphology that is characteristic of ADSCs. The cells were tested for their ability to differentiate into adipocytes, osteocytes, and chondrocytes confirming their mesenchymal properties. When the cells covered the surface of the six-well plate in a confluency of approximately 60%, they were cultivated in differentiation mediums for 30 days. Differentiated cells were positively stained with Alizarin Red, for differentiation to osteocytes, with Oil Red O, for differentiation to adipocytes, and with Alcian Blue for differentiation to chondrocytes (Figure 3). Positive staining of the isolated cells confirms the mesenchymal characteristic of the cells.

## Discussion

ADSCs have been characterized as a cell population with huge potential in different fields of regenerative medicine. Thus, the use of this cell population is rapidly expanding in clinical procedures. Data from clinical and pre-clinical studies show that autogenous ASCs are differentiated after their administration [11,13-15] and have anti-apoptotic, anti-inflammatory, and angiogenic effects [16-18].

The ideal source of MSCs should have the following characteristics: easily and safely accessible, abundant in the human body, requiring minimum manipulation, and yielding large amounts of viable stem cells. Adipose tissue is the only of all human tissues that could meet these traits. Bone marrow is an alternative, but it is a tissue more difficult to obtain, while the harvested cells require special manipulation procedures and cultivation. Numerous researchers have published very good results with bone marrow stem cells, but we should always bear in mind the whole procedure before these stem cells come into use and clinical applications. Obtaining stem cells from bone marrow provides small numbers of cells that need to be incubated and multiplied before being ready to use and this makes impossible their use in one-stage procedures. Bone marrow-derived stem cells have the ability to differentiate into all three lineages - endodermal, mesodermal, and ectodermal- so they could be more useful for a wider range of clinical applications, but clinical reality has proven to be far from the lab results. Concerning adipose tissue, it seems to yield large amounts of MSCs due to its abundance in the human body. Abdominal fat is accessible with subcutaneous procedures, liposuction being the easiest and safest way to collect adipose tissue. Liposuction has the disadvantage of yielding a mixture of tissues, adipose, connective, and blood, that have to be separated and cleaned of the non-useful parts. This means that after collecting the specimen we have to get rid of connective tissue and blood cells in order to process the adipose tissue that harvests the stem cells. This procedure may also be time and money-consuming, depending on the method one should use. There are two main methods of processing the collected tissue via liposuction: enzymatic and mechanic, each of them with several advantages and disadvantages.

Enzymatic methods use enzymes such as collagenase, trypsin, or dispase to dissociate the fatty tissue and release the MSCs. However, they are time-consuming, as they need 30-60 min to digest the tissue and then wash the enzymes out since they must not be directly injected into the patient’s tissues. Furthermore, enzymatic methods are more expensive than the mechanical methods we will analyze further. Finally, the numerous steps such as many centrifuges that are required in these methods increase the manipulation of the stem cells making them probably more vulnerable or ineffective [7,10,19]. On the other hand, a possible advantage of the enzymatic methods is the number of stem cells they yield, but as we mentioned, viability remains a question [3,20]. Throughout any of these steps, the infection of the tissue manipulated is a possible danger. Moreover, laboratory investigations have the final aim of isolating and cultivating or differentiating the cells but very few of them move to the next step of clinical application. Our investigating team, consisting both of laboratory investigators and clinicians (surgeons), focused on bringing the lab results to reality and this was the reason we rejected enzymatic methods for one-stage procedures and clinical applications. In conclusion, it could be argued that enzymatic methods are not suitable for one-stage procedures since they are time-consuming, require specific manipulations, and pose several risks to the tissue of the patient to whom is delivered. On the contrary, we focused on a mechanical method that yields a substantial amount of MSCs within the stromal vascular fraction, with good viability and minimal chances of cells being contaminated through multiple steps of the procedure.Our procedure deals with the challenge of harvesting substantial amounts of MSCs from a single donor and furthermore of maintaining the viability of these cells in order to be used directly, sparing the danger of contamination of the harvested cells and the time-consuming cell proliferation and cultivation that arise from enzymatic methods. Mechanical methods use various mechanical means of processing the adipose tissue, some of them requiring special equipment and others being simple and cheap [3,7,10]. All of them include some basic steps: washing the adipose tissue harvested, homogenization of the tissue and centrifugation in order to obtain the SVF and MSCs [3,7,19,21-25]. Our method, which resembles several steps from different mechanical isolation procedures, manages to isolate MSCs in the stromal vascular fraction by simple and widely used means. The novelty of this article is not the isolation method itself but rather the combination of simple methods and materials that if used properly, can provide a reliable final product of regenerating medicine that can be used immediately and safely. The results of our method did not have the intention to prove just the isolation of MSCs but to produce a final product that contains viable MSCs and can be used in clinical practice widely, requiring no special means. In the first step, a standard liposuction procedure is conducted which is universally accepted and the following emulsification procedure is conducted with an intersyringe procedure, which means that the adipose tissue is “crushed” between two syringes connected with a luer-lock system. This procedure is of great importance and must be carried out gently in order not to cause mechanical damage to the MSCs, destroying their abilities. This simple mechanical method for emulsification spares the need to use any special materials such as microfilters or sonication blenders which are expensive and not widely available. It is of great importance to follow carefully each step in order to have a final clean product. Thorough washing with an isotonic solution removes most of the red blood cells and lidocaine solution that if left in the tissue specimen, will mix with the other cell populations, affecting their isolation and viability. Further mincing of the tissue and removing any large stringy pieces of connective tissue is also essential because if not removed they will decant with the stem cells to the bottom and prevent us from having a clear aspiration of pure cells. Finally, the emulsification intersyringe procedure has to be done very gently so as not to harm and destroy the stem cells that lie between the adipose cells. Bearing all these in mind, our method is cheap, fast, and reliable by the use of simple means, compared to other mechanical isolation methods. The cell population yielded by this method varies in each tube, but in a total amount of 20ml of finally processed tissue the overall cell population is satisfying and so is cell viability. Our results are consistent with several other publications that mention satisfying MSC populations and excellent viability by use of mechanical methods. The amount of time needed to process the adipose tissue obtained is relatively short. The procedure of liposuction-emulsification-centrifugation of the adipose tissue ranges between some minutes (15-30) and depends mainly on the time needed for the sedimentation of the ASCs. The manipulation of the harvested tissue is minimal and can be done inside the same operating room where the liposuction takes place. The speed and simplicity of the whole procedure, the number of ASCs, and their viability at the end of the process make our method ideal for one-stage procedures. All the means can be readily accessible in the operating theatre and the presence of a centrifuge in the operating room also can be easily managed in a hospital. The procedure is completely safe since it uses autologous tissue and the stem cells are not processed by any chemical or enzymatic means. Since there is no allogeneic tissue transplantation, no special permissions are needed and autologous stem cells could be readily used by the patient for various purposes in a wide range of procedures such as general surgery (perianal fistulas, chronic wounds), orthopedics, or dentistry. 

## Conclusions

Mechanical methods for processing adipose tissue and obtaining MSCs are safe, cost-effective, and suitable for one-stage procedures (e.g. delivering stem cells in perianal fistulas), favoring patients’ comfort and health care system expenses. The intersyringe procedure and centrifugation steps for obtaining SVF and MSCs that we describe here are effective, quick, safe, and can be reproduced in most laboratories or health care units, as no special equipment is needed for cell isolation. Therefore, the cost-effectiveness and simplicity of this method render it ideal for widespread use in a range of surgical procedures.
